# The generation and use of animal models of osteosarcoma in cancer research

**DOI:** 10.1016/j.gendis.2022.12.021

**Published:** 2023-03-24

**Authors:** Feifei Pu, Haoyu Guo, Deyao Shi, Fengxia Chen, Yizhong Peng, Xin Huang, Jianxiang Liu, Zhicai Zhang, Zengwu Shao

**Affiliations:** a Department of Orthopedics, Wuhan Hospital of Traditional Chinese and Western Medicine (Wuhan No.1 Hospital), Tongji Medical College, Huazhong University of Science and Technology, Wuhan, Hubei 430022, China; b Department of Orthopedics, Union Hospital, Tongji Medical College, Huazhong University of Science and Technology, Wuhan, Hubei 430022, China; c Department of Radiation and Medical Oncology, Zhongnan Hospital, Wuhan University, Wuhan, Hubei 430071, China; d Hubei Cancer Clinical Study Center, Zhongnan Hospital, Wuhan University, Wuhan, Hubei 430071, China

**Keywords:** Animal model, Cancer, Osteosarcoma, Pathogenic mechanism, Translational research

## Abstract

Osteosarcoma is the most common malignant bone tumor affecting children and adolescents. Currently, the most common treatment is surgery combined with neoadjuvant chemotherapy. Although the survival rate of patients with osteosarcoma has improved in recent years, it remains poor when the tumor(s) progress and distant metastases develop. Therefore, better animal models that more accurately replicate the natural progression of the disease are needed to develop improved prognostic and diagnostic markers, as well as targeted therapies for both primary and metastatic osteosarcoma. The present review described animal models currently being used in research investigating osteosarcoma, and their characteristics, advantages, and disadvantages. These models may help elucidate the pathogenic mechanism(s) of osteosarcoma and provide evidence to support and develop clinical treatment strategies.

## Introduction

Osteosarcoma (OS) is the most common malignant bone tumor affecting children and adolescents.^1^^,^^2^ Current treatments for OS involve surgical resection of the affected area and multi-agent chemotherapy, although the survival rate is generally poor for those with ongoing metastases.^3^ Because the treatment for OS has remained unchanged for the past few decades, there is a need for further advances in the understanding of OS biology and therapeutics.^4^ A major characteristic of OS is its heterogeneity, both at the intra-tumoral level and also between individuals. Therefore, the common genomic initiating biological processes driving osteosarcomagenesis are still not identified. The complexity of the somatic genome of OS is a major cause of intra-tumoral heterogeneity and is characterized by chromosomal aneuploidy, alteration of genes by mutation and/or variation of copy number, with genomic instability featured by massive rearrangement through chromothripsis, and the presence of patterns of localized hypermutated regions, named Kataegis.^5^

Reliable animal models can accurately replicate the disease.^6^ To investigate pathogenesis, diagnosis, treatment, and drug screening, the establishment of animal models closely replicating the biological behavior and pharmacokinetics of human OS is an effective method to develop effective treatment strategies for OS.^7^, ^8^, ^9^, ^10^ In the present review, we describe various animal models currently being used in research investigating OS, their characteristics, and advantages and disadvantages. This may help elucidate the mechanism(s) of OS and provide evidence to support and develop clinical treatment strategies.

## Classification of animal models

**Figure 1:**
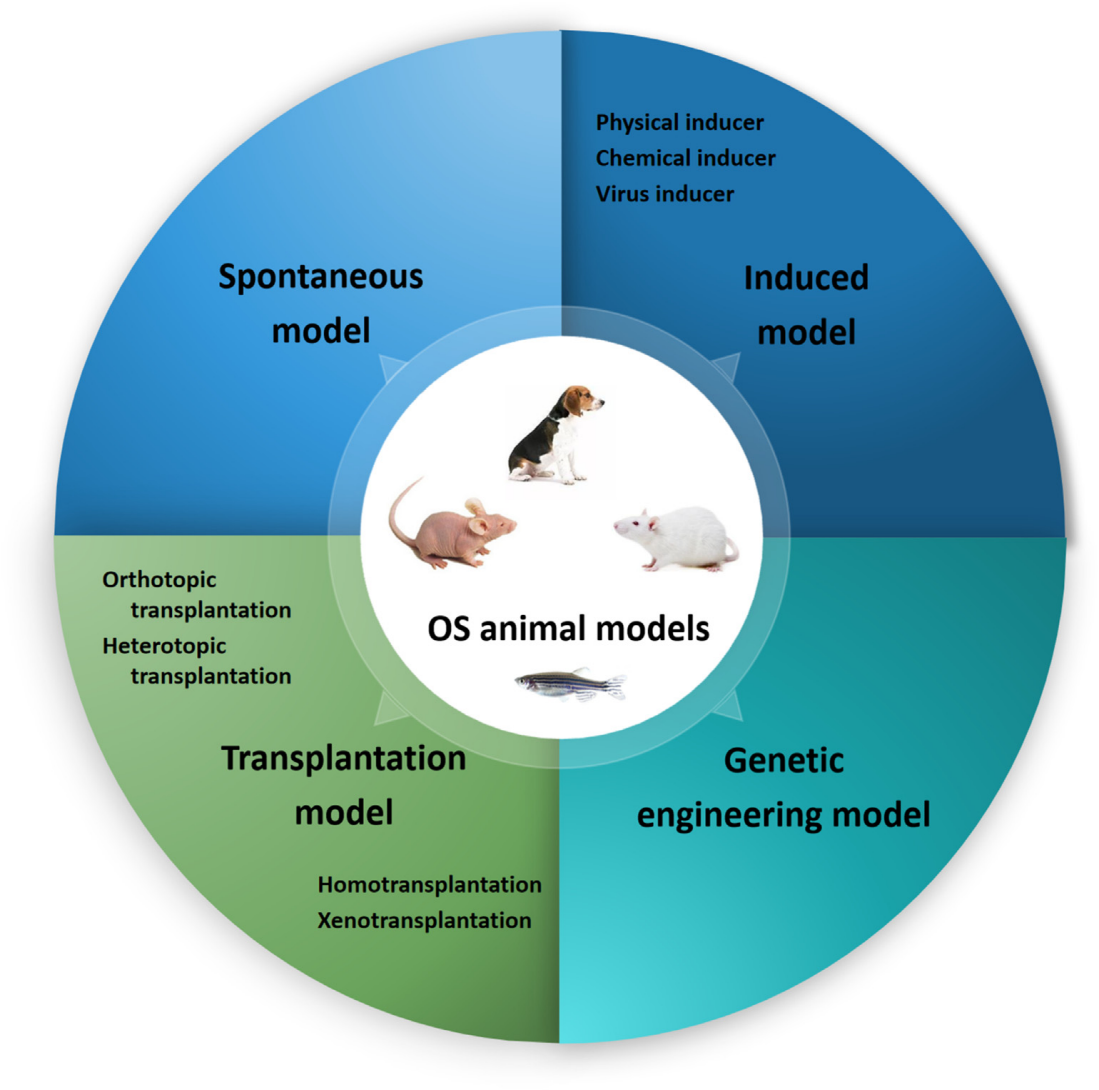
Classification of animal models of osteosarcoma.

Cancer models involving experimental animals can be roughly divided into four categories: spontaneous, induced, genetically engineered, and transplant (Fig. 1).^10^ Spontaneous OS models occur naturally or are caused by genetic mutations in experimental animals without any intentional artificial treatment, and most commonly involve dogs and mice.^11^^,^^12^ The induced OS model involves the induction of cancer using physical mutagens, chemical agents, or viruses.^13^ Genetically engineered OS animal models are used to investigate the function and mechanism of specific genes in primary or metastatic OS by inoculating transgenic cancer cells into the bone marrow cavity of animals or creating animal models by inoculating cancer cells into the bone marrow cavity of transgenic animals.^14^, ^15^, ^16^ Transplantable OS animal models involve the inoculation of cancer cells into the bone marrow cavity or other specific parts of experimental animals to induce cancers. This method has a high value in studies investigating metastatic OS.^17^^,^^18^

## Spontaneous OS model

The spontaneous tumor model is similar to the human OS in terms of biological characteristics, including genetic and environmental factors, which makes it an appropriate choice for investigating the etiology and pathology of OS.^19^^,^^20^ Spontaneous OS is much more common in large dogs than in humans, making the dog an attractive candidate model to study human disease.^20^ Canine OS is indistinguishable from human tumors at the histological and gene expression levels. Many of the genes involved in human OS pathogenesis appear to participate in canine OS, including P53, RB, and PTEN.^21^, ^22^, ^23^ Although canine OS serves as an excellent comparative tumor model for human OS, there are some limitations to be considered. First, OS affects skeletally mature, geriatric dogs, which is different from humans where the peak of incidence occurs during adolescence. Second, some breeds have specific heritable germ-line mutations in certain genes that may influence OS biology, progression, and response to treatment without driving the initiation of the disease.^7^ A recent study validated a spontaneous canine OS model for human disease by evaluating the expression of driving genes and immunohistochemical markers known to be important in human OS. The findings were similar to those described previously for human OS, which suggests that canine OS may represent a useful model for the study of the human form of the disease.^24^ Another study found that co-targeting of DNA, RNA, and protein molecules can result in optimal outcomes for treating OS and pulmonary metastasis in spontaneous and experimental metastasis mouse models.^25^ Currently, however, the spontaneous OS model is rarely used due to several drawbacks, including unstable output caused by multiple factors, a prolonged period of tumor formation, and poor homogeneity, which makes it difficult to conduct comparative studies on a regular basis.^26^^,^^27^ In addition, OS occurs mostly in children and adolescents, while it is different in animal models^28^; more specifically, OS is more prevalent among middle and old-age canids and, once it occurs, it progresses rapidly.^29^, ^30^, ^31^

## Induced OS model

The induced cancer model refers to directly or indirectly exposing specific parts of animals to carcinogenic factors to induce cancer in targeted organs.^32^^,^^33^ This method eventually results in a high rate of carcinogenesis using simple procedures, making it easy to establish OS model animals.^34^^,^^35^ However, the effect of carcinogenic factors is not completely under control, which may lead to low repeatability (*i.e.*, producing different types of tumors each time), thus disrupting the tumor microenvironment and affecting cancer development.^36^^,^^37^

## Physical induction

Radionuclides are strong carcinogens, and virtually all radionuclides of osteotactic nature can cause OS when animals are exposed.^35^, ^36^, ^37^, ^38^ OS is induced by radionuclides either by injecting a saline solution of the radionuclide into the animal or by irradiating the animal directly with the radionuclide.^39^ When a nuclide salt solution is used to induce cancer, the dose should be appropriate. It is difficult to induce cancer when the dose is too low, while too much nuclide can kill cancer cells, which leads to the failure of the experiment.^40^ Tinkey et al observed induction of OS in Sprague–Dawley rats 4–8 months after exposing the hind legs of the animals to ^60^Co γ rays.^41^ In addition, some studies have reported that radionuclides, including ^241^Am, ^239^Pu, ^238^Pu, and ^237^Np, can also induce OS, although the cancer rate is not 100%.^42^, ^43^, ^44^ On the whole, these models yielded tumors that histologically resembled human cancer and produced cell lines that complement human OS studies. Despite the high penetrance of the models, their relevance remains unclear since the majority of OS in humans is sporadic, while the carcinogen-induced murine model is more representative of a therapy-induced disease.^7^

## Chemical induction

Injecting chemical agents into the muscles of animals at a constant concentration can also induce OS, but usually takes a long time (typically >40 weeks).^45^ These chemicals include beryllium zinc silicate, aflatoxin B1, arsenite, 7,12-dimethylbenzanthracene, 4-hydroxyaminoquinoline-1-oxide, beryllium oxide, methylcholanthracene, N-hydroxyl, copper compound of 2-acetamide fluorene, and diethylnitrosamine.^46^^,^^47^ However, the chemical-induced cancer model has low reproducibility, and the chemicals can be harmful to researchers. As such, to date, this method has rarely been used to generate animal models. It is undeniable that the auxiliary effect of chemical factors can increase the tumorigenesis rate of cancer-inducing and accelerate the experimental process. Cancer-promoting agents are involved in the formation of tumors through the carcinogenic effect of carcinogenic agents. Carcinogenic agents cause normal cells to become cancerous, while cancer-promoting agents cause cancerous cells to proliferate.^7^^,^^48^ Therefore, the chemical-induced OS model is of great significance in the fields of tumorigenic factor screening, epidemiological investigation, identification of high-risk tumor populations, and exploration of tumorigenic susceptibility genes.

## Virus induction

With advances in molecular biology and virology, some viruses have been found to exert carcinogenic effects.^49^^,^^50^ By integrating with the host cell genome, viruses activate proto-oncogenes and induce tumorigenesis.^51^, ^52^, ^53^ Such viruses are known as cancer viruses. Viruses that have been used to induce OS formation include SV40, Moloney sarcoma virus (MSV), FBJ, RFB, and FBR OS.^54^, ^55^, ^56^, ^57^, ^58^, ^59^ As a double-stranded DNA virus, SV40 can induce OS when injected into newborn hamsters.^55^ Olson et al induced New Zealand rats using the Moloney sarcoma virus and found that the formation rate of cancer was >80%, and the histological morphology of the induced tumor was similar to that of human OS.^60^ Finkel et al successfully induced an OS model by injecting cancer cell-removed extract (containing virus) into neonatal mice.^61^ In general, virus-induced OS models take less time than physical and chemical-induced OS models and are more reproducible.^62^ Taking MSV as an example, after the virus strain was injected into the bone marrow cavity of the proximal tibia of neonatal rats, a significant mass appeared at the injection site within 14 days, which resembled human OS histologically. Most rats died within 13–21 days, and lung metastases usually occurred within 4 weeks of injection.^63^

## Cancer transplantation model

Cancer transplantation involves the transplantation of tumor tissue (mass or cell line) to specific sites of model animals.^64^ Once the primary tumor is formed, it can be transplanted into the next generation(s) of model animals.^65^ The transplanted animal model of OS is currently one of the most popular methods because it has many advantages, including clear and stable tumor characteristics, a short experimental period, and a high tumor formation rate.^66^, ^67^, ^68^ This model remains very useful for studies investigating drug screening, treatment, invasion, and metastasis of OS.^19^ The principal limitation is that the approach uses fully developed OS cells and therefore does not provide information about the initiation of the tumor and its etiology. Furthermore, since the tumor microenvironment can contribute significantly to the tumor behavior, such interactions may be lost when establishing the disease by direct introduction into a recipient animal.^22^ In certain circumstances, the injected cell line may not be metastatic in the rodent context, making it impossible to study the dissemination of the disease. Despite these limitations, many groups have successfully used this model to identify factors involved in OS migration and more importantly for screening drugs with tumoricidal potential.^23^ Cancer transplantation can be classified as homotransplantation or xenotransplantation, according to whether a tumor tissue block or cell line is transplanted into homogenous or allogeneic animals.^69^, ^70^, ^71^, ^72^, ^73^ It can also be divided into ectopic and orthotopic transplantation, according to whether the tumor tissue is transplanted to the site corresponding to the primary tumor or other anatomical locations in the recipient animals.^74^, ^75^, ^76^, ^77^

## Homotransplantation

Allotransplantation is the transplantation of a tumor tissue block or cell line into an allogeneic or homologous animal.^78^ For newborn or immunodeficient animals, the transplant success rates are satisfactory, while mature animals have a relatively low rate due to the effects of their immune systems.^79^ Sottnik et al inoculated the luciferase-transfected murine OS cell line DLM8 into the medullary cavity of the proximal tibia of 6-to-8-week-old female C3H mice and successfully established an animal model of OS *in situ*. The earliest time of lung metastasis in all C3H mice was 16 days after inoculation, and the median survival of all cancer-bearing mice was 33 days.^27^

## Xenotransplantation

Xenotransplantation involves the transplantation of tumor tissue or a cell line into another animal species; however, early xenotransplantation had a low success rate due to rejection.^80^ Some researchers transplanted human OS cells into mice after suppressing the immunity of adult mice with high-dose X-ray irradiation and immunosuppressive agents.^81^ Floersheim et al introduced a method of short-term immunosuppression, in which host mice were treated with methylbenzazide, cyclophosphamide, and anti-lymphocyte serum for 4–6 days, then human OS tumor tissue was transplanted subcutaneously into mice, which achieved a tumorigenic rate of 100%.^82^ Guo et al inoculated the human OS cell line 143 B transfected with pcDNA3.1 plasmid and dNLRP5 into the left tibial medullary cavity of 4-week-old NCR-nu/nu nude mice to establish the xenograft animal model of OS and studied the anti-cancer and anti-metastatic activities of dNLRP5 *in vivo*.^83^

## Heterotopic transplantation

Heterotopic transplantation refers to the transplantation of OS cells or tissue blocks into sites other than bone, including subcutaneous and venous grafts.^84^ Metastasis is rarely observed in animals that have undergone subcutaneous injections. However, Ory et al successfully established a lung metastatic model of OS in C3H mice by intravenously injecting 0.05 mL of the POS-1 cell line at a concentration of 10^5^/mL; all tumor-bearing mice died after 3 weeks.^85^ The principal limitation is that the microenvironment of heterotopic tumors is different from the natural status, which limits its further applications in studying etiology and immunology.

## Orthotopic transplantation

Orthotopic transplantation refers to an animal model of OS constructed by inoculating human OS cells or tissue blocks into the organs corresponding to the original site.^86^ In recent years, orthotopic transplantation has attracted increasing attention with the deepening understanding of OS animal models and the biological characteristics of OS cells. Compared with heterotopic transplantation, orthotopic transplantation of OS is more suitable for the establishment of OS animal models owing to its short incubation period, rapid growth, and high metastasis rate.^87^, ^88^, ^89^ Within a relatively native context, orthotopic transplantation enables the investigation of primary OS formation as well as metastatic progression, thereby replicating the entire spectrum of biological behavior of OS.^90^ Miretti et al selected highly tumorigenic KSL cells and injected the cell suspension directly into the distal femur of nude mice; tumors formed in all mice, with rapid tumor growth and good tumor cell properties.^91^ However, the complexity of orthotopic injection and operation restricts the reproducibility of transplantation.

## Genetically engineered models

With the development of epigenetics, it has been shown that the occurrence and development of cancers are accompanied by changes in the expression of many oncogenes and tumor suppressor genes.^92^^,^^93^ Transgenic OS animal models can adequately simulate the physiological and pathological state of the human body and have good consistency with the occurrence and development process of OS.^94^ Moreover, genetic engineering technology can also simulate precancerous lesions, which is beneficial in revealing the molecular mechanism(s) of tumors and provides new directions for human OS research.^95^ Therefore, transgenic animal models can simultaneously exhibit gene expression and phenotypic effects from the perspectives of time and space at the overall level. These OS-related genes include *p53*, *RB*, *C-FOS*, *TWIST*, *p1*4ARF, *p16INK4a*, *NF2*, *p27*, *PRKAR1A*, and *p21CIP*.^96^, ^97^, ^98^, ^99^, ^100^, ^101^ These genes belong to the family of cancer suppressor genes, except *C-FOS* and *TWIST*. Silencing cancer suppressor genes and enhancing the expression of oncogenes are commonly used methods to construct transgenic models; among them, silencing *p53* and *RB* is often used to construct OS models.^96^ Experiments have confirmed that mouse *p53* gene silencing can induce the occurrence of OS, suggesting that *p53* mutation plays an important role in the induction of OS.^102^ Although silencing *RB* alone does not induce OS, co-silencing *RB* and *p53* can significantly accelerate cancer development.^96^ However, transgenic animal models also have shortcomings. Approximately 85% of OS models induced by genetic engineering occur in the axial bone (jaw, ribs, vertebrae, skull, and sternum), and only 16% occur in the limb bones, which does not accurately reflect human OS.^103^ As such, transgenic animal models require further optimization. The success rate of building OS animal models may be improved by silencing cancer suppressor genes, increasing the expression of multiple oncogenes, or simultaneously silencing cancer suppressor genes and increasing the expression of oncogenes.

## Establishment of animal models of OS

### Animal choice

Presently, the selection of experimental model animals for OS tends to include dogs, rats, and mice.^23^^,^^104^, ^105^, ^106^ Dogs can spontaneously develop OS in a form similar to humans and are often used as spontaneous cancer models.^107^ Mice have the same organ systems as humans and share a high degree of genetic similarity.^102^ Nude mice, a type of immunodeficient animal, are widely used as cancer animal models because they are easy to feed and manage.^108^ Nude mice can be inoculated with cancer cells from different sources, making xenotransplantation possible.^109^ Severe combined immunodeficiency (SCID) mice are deficient in both humoral and cellular immunity, and can be used for xenograft animal models for cancer cells in nude mice after model failure.^110^^,^^111^ Joseph et al reported that immunoactive mice, such as C3H, were better than nude mice in xenotransplantation of murine-derived tumors; the selection of immunoactive mice does not disrupt the interaction between cancer and host microenvironment, and it is more conducive to the evaluation of drug efficacy.^112^ Compared with mice, Sprague–Dawley and Wistar rats are larger in size, easier to operate on and yield more tissue. Therefore, tumor-bearing models are often prepared in batches for studying cancer chemotherapy and immunotherapy. In addition, zebrafish xenotransplantation has also been used to study the role of specific genes in recent years due to its rapid OS model construction.^9^^,^^113^ Although zebrafish appear to be an appealing model to investigate OS due to its similarities with human osteogenesis, only a few OS-specific studies have been conducted.^114^^,^^115^ Regarding etiology, the high degree of genetic similarity between zebrafish and human cancers indicates that affected regions are evolutionarily conserved.^116^ Therefore, as a rapid model system, zebrafish enable the investigation of multiple candidate gene defects.

### Transplanted OS cells

Transplanted cell lines can be divided into lines of murine or human origin. Mice-derived cell lines mainly include Dunn, K7, K8, K12, K14, K37, and UMR106-01, which are mostly used for homotransplantations, whereas human cell lines mainly include U2OS, TE85, HOS, MNNG, KRIB, 143 B, and SaOS-2, which are mostly used for xenotransplantation.^117^^,^^118^ Different transplanted cells can influence the cancer formation rate and lung metastasis rate in animal cancer models. Yuan et al transplanted seven different osteosarcoma cell lines (G292, MG-63, TE85, U2OS, SaOS-2, 143 B, and SaOS-LM7) *in situ* into the tibial medullary cavity of NOD/SCID mice at the same cell concentration and found that neither G292 nor TE85 developed tumor or lung metastasis. The lung metastasis rate of U2OS cells was only 1/7. The tumor formation rates of MG-63 and SaOS-2 cells were 2/7 and 4/7, respectively; however, no lung metastasis occurred. The tumor formation rate of 143 B cells was 100%, and the lung metastasis rate was 87.5%. The tumor formation rate of SaOS-LM7 was 100%, and the lung metastasis rate was 50%.^119^

### Inoculation methods

The raw material for cancer transplantation includes two forms, cancer cell suspension, and primary tumor tissue.^120^, ^121^, ^122^, ^123^ Cell suspension inoculation has the advantages of simple procedure(s), low cost, and good reproducibility. However, cells in suspension are relatively dispersed, and the interaction among cancer cells is weakened or destroyed, making cancer cells easily cleared by the body's immune system.^121^ Moreover, cancer cells treated with enzymes before transplantation may lose their primeval properties, which may alter the biological characteristics of cancer, including tumor formation rate and metastatic capacity.^124^ Cell suspensions are mostly inoculated orthotopically in the bone marrow; however, the medullary cavity of the lower femur in nude mice is narrow, which easily leads to suspension overflow of the bone marrow and insufficient number of cancer cells in the medullary cavity.^76^ Compared with cell suspension injection, the method of tissue block transplantation appears to be significantly superior and yields a higher tumor formation rate, faster tumor growth, more invasiveness of the bone cortex and soft tissue, and a higher metastasis rate.^125^ Therefore, tissue block transplantation can be used as a reliable method to establish *in situ* OS animal models.

## Application of OS animal models

### Morphological observation of cancer

The dynamic observation of transplanted tumors in animal models includes measuring size, location, and color, and assessing histological characteristics, animal behavior, weight, vital signs, and histological sections of important organs (*i.e.*, heart, liver, spleen, lungs, and kidneys).^126^ These indicators are helpful for further understanding the mechanism of OS formation, expansion, invasion, and metastasis.

### Investigating the etiology of OS

The etiology of OS remains unclear, and studying the causes of OS is one of the primary functions of animal models.^127^ Dogs can develop OS spontaneously and are similar to humans in terms of etiology, clinical manifestations, pathological imaging features, metastatic rate and site, and response to treatment.^128^ Therefore, dogs are ideal models for studying the causes of OS. The loss of cancer suppressor function by mutation of the *p53* gene is often the cause of human malignancies, including OS.^18^^,^^96^ Using dogs with spontaneous OS as the research model, Leeuwena et al analyzed the relationship between *p53* gene mutation and OS incidence, and found that all tumor-harboring dogs had *p53* gene mutations. The authors also found that the mutation frequency was similar to that of humans.^129^ Johnson et al used single-stranded conformational polymorphism to study the role of *p53* in the occurrence of human and canine OS and found that the mutation sites and types of *p53* gene were virtually identical.^130^

### Mechanism of distant metastasis of OS

Metastasis is the most common cause of death due to OS, especially in those who develop lung metastasis. Lung metastasis occurs in approximately 20% of OS patients and is strongly associated with lower survival rates.^131^ As such, it is highly valuable to study the mechanism(s) of metastasis. In the process of observing lung metastasis in animal models of OS, most studies still use lung specimens obtained from tumor-bearing animals for various tests.^132^ With the development of fluorescence markers and luciferase imaging technology, the sensitivity and accuracy of *in vivo* detection have been improved.^133^ Sottnik et al transfected RSV-pGL4.17, a plasmid containing the luciferase gene, into the murine OS cell line DLM8 using electroporation technology, and then collected the transfected DLM8 for orthotopic transplantation. After the intraperitoneal injection of luciferin, an IVIS100 imaging system (PerkinElmer, Waltham, MA, USA) was used for observation. Subsequently, a series of new methods for non-invasive dynamic follow-up monitoring of lung metastasis was proposed.^27^ Silvia et al injected K5L-Luc cells expressing both green fluorescent protein and luciferase protein into the femoral end of BALB/c nude mice and monitored lung metastasis using an *in vivo* imaging system. On day 38, the first case of lung metastasis was observed, followed by lung metastasis in all mice for 3 weeks.^91^

### Evaluation of therapeutic efficacy

Guo et al studied the antitumor activity of the dominant-negative LRP5 receptor (DNLRP5) *in vivo* by using an animal model constructed using the 143 B cell line and found that DNLRP5 inhibited cancer growth and metastasis in model animals by blocking the Wnt signaling pathway and, at the same time, decreased the expression of related markers in cancer cells.^83^ Sottnik et al established an *in situ* murine OS model with spontaneous metastasis and found that doxorubicin and carboplatin significantly delayed lung metastasis in model animals.^27^ The anticancer properties of two different quinoline–platinum complexes on *in vitro* (2D and 3D cultured cells) and *in vivo* (xenograft tumor: human OS in mice) models were reported and highlighted the importance of chelation in antitumor properties, suggesting that the [PtCl (8-O-quinoline) (dmso)] (2) may be a promising agent for the treatment of human OS cancers resistant to cisplatin.^134^ It is worth mentioning that the chorioallantoic membrane (CAM) model is particularly interesting as the chick embryo is not considered to be a living animal until day 17 of development in most countries and therefore does not fall under animal experiment. Guder et al analyzed the drug sensitivity of human high-grade OS in a chick CAM model, and they prove that analysis of drug sensitivity is possible on the CAM and that the clinical applicability is justified.^135^ In the study of preclinical justification of pbi-shRNA EWS/FLI1 lipoplex (LPX) treatment for Ewing's sarcoma, toxicology studies in mini-pigs provide the justification to initiate clinical testing.^136^

## Conclusions

**Figure 2:**
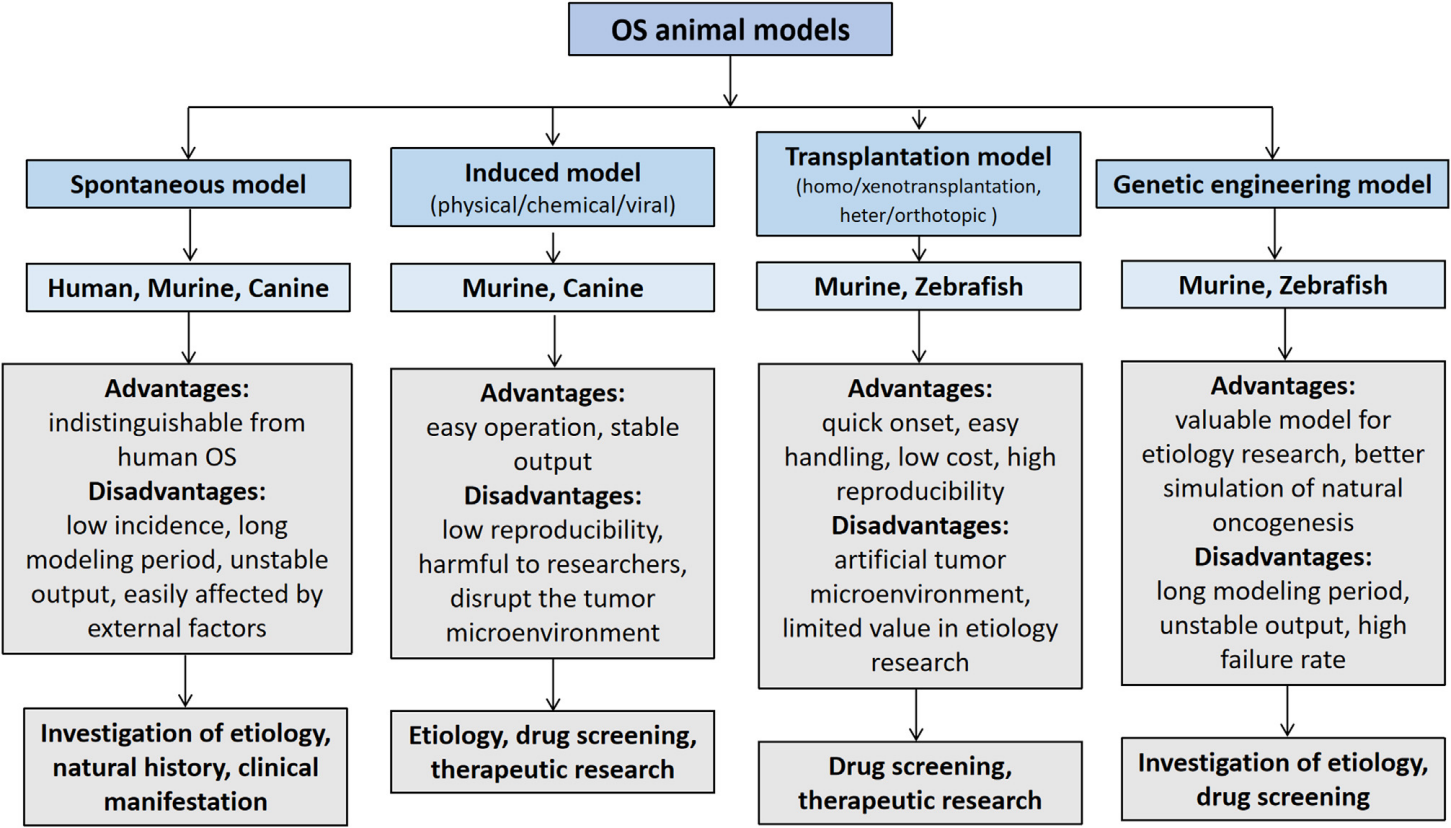
Advantages and disadvantages of various animal models of osteosarcoma.

Animal models of OS have various advantages and disadvantages (Fig. 2). Ideal animal models are highly valuable for understanding the occurrence and development of OS, as well as the research and development of new drugs and therapeutic methods/strategies. The construction of animal models provides new ideas and solutions for the study of OS and, to a certain extent, highlights the biological characteristics of human OS, which helps explore pathogenesis, metastasis, and drug-resistance mechanisms. With advances and developments in molecular biology and genetic engineering, an increasing number of transgenic animal models have been used in tumor research to further reveal the molecular mechanisms of tumors. In the future, more advanced comparative animal models for human OS will be developed, laying a solid foundation for the final conquest of OS.

## Conflict of interests

The authors declare that they have no conflict of interests.

## Funding

This work was supported by the 10.13039/501100001809National Natural Science Foundation of China (No. 82274559, 81904231, 82072978, 82072979), the 10.13039/501100002858China Postdoctoral Science Foundation (No. 2020M672369), and the 10.13039/501100003819Natural Science Foundation of Hubei Province, China (No. 2020CFB861).

## References

[1] Eaton B.R., Schwarz R., Vatner R. (2021). Osteosarcoma. Pediatr Blood Cancer.

[2] Isakoff M.S., Bielack S.S., Meltzer P. (2015). Osteosarcoma: current treatment and a collaborative pathway to success. J Clin Oncol.

[3] Harrison D.J., Geller D.S., Gill J.D. (2018). Current and future therapeutic approaches for osteosarcoma. Expert Rev Anticancer Ther.

[4] Chen C., Xie L., Ren T. (2021). Immunotherapy for osteosarcoma: fundamental mechanism, rationale, and recent breakthroughs. Cancer Lett.

[5] Corre I., Verrecchia F., Crenn V. (2020). The osteosarcoma microenvironment: a complex but targetable ecosystem. Cells.

[6] Peng Y., Qing X., Shu H. (2021). Proper animal experimental designs for preclinical research of biomaterials for intervertebral disc regeneration. Biomater Transl.

[7] Guijarro M.V., Ghivizzani S.C., Gibbs C.P. (2014). Animal models in osteosarcoma. Front Oncol.

[8] Fan T.M. (2010). Animal models of osteosarcoma. Expert Rev Anticancer Ther.

[9] Mohseny A.B., Hogendoorn P.C.W. (2014). Zebrafish as a model for human osteosarcoma. Adv Exp Med Biol.

[10] Jarvis S., Koumadoraki E., Madouros N. (2021). Non-rodent animal models of osteosarcoma: a review. Cancer Treat Res Commun.

[11] Nascimento-Gonçalves E., Mendes B.A.L., Silva-Reis R. (2021). Animal models of colorectal cancer: from spontaneous to genetically engineered models and their applications. Vet Sci.

[12] Nogueira A., Pires M.J., Oliveira P.A. (2017). Pathophysiological mechanisms of renal fibrosis: a review of animal models and therapeutic strategies. In Vivo.

[13] Osum S.H., Watson A.L., Largaespada D.A. (2021). Spontaneous and engineered large animal models of neurofibromatosis type 1. Int J Mol Sci.

[14] Sampson V.B., Kamara D.F., Kolb E.A. (2013). Xenograft and genetically engineered mouse model systems of osteosarcoma and Ewing's sarcoma: tumor models for cancer drug discovery. Expet Opin Drug Discov.

[15] Alegre F., Ormonde A.R., Snider K.M. (2018). A genetically engineered microRNA-34a prodrug demonstrates anti-tumor activity in a canine model of osteosarcoma. PLoS One.

[16] Lahr C.A., Landgraf M., Wagner F. (2022). A humanised rat model of osteosarcoma reveals ultrastructural differences between bone and mineralised tumour tissue. Bone.

[17] Jacques C., Renema N., Ory B. (2019). Murine models of bone sarcomas. Methods Mol Biol.

[18] Walkley C.R., Qudsi R., Sankaran V.G. (2008). Conditional mouse osteosarcoma, dependent on p53 loss and potentiated by loss of Rb, mimics the human disease. Genes Dev.

[19] Dass C.R., Choong P.F.M., Ek ETH (2007). Human xenograft osteosarcoma models with spontaneous metastasis in mice: clinical relevance and applicability for drug testing. J Cancer Res Clin Oncol.

[20] Maloney C., Edelman M.C., Kallis M.P. (2018). Intratibial injection causes direct pulmonary seeding of osteosarcoma cells and is not a spontaneous model of metastasis: a mouse osteosarcoma model. Clin Orthop Relat Res.

[21] Weinman M.A., Ramsey S.A., Leeper H.J. (2021). Exosomal proteomic signatures correlate with drug resistance and carboplatin treatment outcome in a spontaneous model of canine osteosarcoma. Cancer Cell Int.

[22] Luu H.H., Kang Q., Park J.K. (2005). An orthotopic model of human osteosarcoma growth and spontaneous pulmonary metastasis. Clin Exp Metastasis.

[23] Chaffee B.K., Allen M.J. (2013). A clinically relevant mouse model of canine osteosarcoma with spontaneous metastasis. In Vivo.

[24] Al-Khan A.A., Gunn H.J., Day M.J. (2017). Immunohistochemical validation of spontaneously arising canine osteosarcoma as a model for human osteosarcoma. J Comp Pathol.

[25] Jian C., Tu M.J., Ho P.Y. (2017). Co-targeting of DNA, RNA, and protein molecules provides optimal outcomes for treating osteosarcoma and pulmonary metastasis in spontaneous and experimental metastasis mouse models. Oncotarget.

[26] Brady J.V., Troyer R.M., Ramsey S.A. (2018). A preliminary proteomic investigation of circulating exosomes and discovery of biomarkers associated with the progression of osteosarcoma in a clinical model of spontaneous disease. Transl Oncol.

[27] Sottnik J.L., Duval D.L., Ehrhart E.J. (2010). An orthotopic, postsurgical model of luciferase transfected murine osteosarcoma with spontaneous metastasis. Clin Exp Metastasis.

[28] Yu X., Yustein J.T., Xu J. (2021). Research models and mesenchymal/epithelial plasticity of osteosarcoma. Cell Biosci.

[29] Rodriguez C.O. (2014). Using canine osteosarcoma as a model to assess efficacy of novel therapies: can old dogs teach us new tricks?. Adv Exp Med Biol.

[30] Mason N.J. (2020). Comparative immunology and immunotherapy of canine osteosarcoma. Adv Exp Med Biol.

[31] Beck J., Ren L., Huang S. (2022). Canine and murine models of osteosarcoma. Vet Pathol.

[32] Halazonetis T.D., Gorgoulis V.G., Bartek J. (2008). An oncogene-induced DNA damage model for cancer development. Science.

[33] Neufert C., Becker C., Neurath M.F. (2007). An inducible mouse model of colon carcinogenesis for the analysis of sporadic and inflammation-driven tumor progression. Nat Protoc.

[34] Komura S., Semi K., Itakura F. (2016). An *EWS-FLI1*-induced osteosarcoma model unveiled a crucial role of impaired osteogenic differentiation on osteosarcoma development. Stem Cell Rep.

[35] Cobb L.M. (1970). Radiation-induced osteosarcoma in the rat as a model for osteosarcoma in man. Br J Cancer.

[36] Czitrom A.A., Pritzker K.P., Langer F. (1976). Virus-induced osteosarcoma in rats. J Bone Joint Surg Am.

[37] Rosemann M., Kuosaite V., Nathrath M. (2002). The genetics of radiation-induced osteosarcoma. Radiat Protect Dosim.

[38] Ingleton P.M., Underwood J.C., Hunt N.H. (1977). Radiation induced osteogenic sarcoma in the rat as a model of hormone-responsive differentiated cancer. Lab Anim Sci.

[39] Acaroğlu R.E., Gögüs T., Ercan M.T. (1995). Experimental induction of osteosarcoma by subperiosteal radioactive phosphorus injections in rats. Nucl Med Biol.

[40] Allouche M., Delbrück H.G., Klein B. (1980). Malignant bone tumours induced by a local injection of colloidal radioactive 144 Cerium in rats as a model for human osteosarcomas. Int J Cancer.

[41] Tinkey P.T., Lembo T.M., Evans G.R. (1998). Postirradiation sarcomas in Sprague-Dawley rats. Radiat Res.

[42] Raabe O.G. (1984). Comparison of the carcinogenicity of radium and bone-seeking actinides. Health Phys.

[43] Lord B.I., Austin A.L., Ellender M. (2001). Tumorigenic target cell regions in bone marrow studied by localized dosimetry of 239Pu, 241Am and 233U in the mouse femur. Int J Radiat Biol.

[44] Ellender M., Harrison J.D., Pottinger H. (2001). Induction of osteosarcoma and acute myeloid leukaemia in CBA/H mice by the alpha-emitting nuclides, uranium-233, plutonium-239 and amercium-241. Int J Radiat Biol.

[45] Taguchi S., Kuriwaki K., Souda M. (2006). Induction of sarcomas by a single subcutaneous injection of 7, 12-dimethylbenz[a]anthracene into neonatal male Sprague-Dawley rats: histopathological and immunohistochemical analyses. Toxicol Pathol.

[46] Sieber S.M., Correa P., Dalgard D.W. (1979). Induction of osteogenic sarcomas and tumors of the hepatobiliary system in nonhuman primates with aflatoxin B1. Cancer Res.

[47] Bersch V.P., Osvaldt A.B., Edelweiss M.I.A. (2009). Effect of nicotine and cigarette smoke on an experimental model of intraepithelial lesions and pancreatic adenocarcinoma induced by 7, 12-dimethylbenzanthracene in mice. Pancreas.

[48] Robinson N.B., Krieger K., Khan F.M. (2019). The current state of animal models in research: a review. Int J Surg.

[49] An S., Liao L., Lin Y. (2021). Widespread hydrogen bonding in the proteins of HIV-1 may confer carcinogenic risks to AIDS patients. DNA Repair.

[50] Adly H.M., Saleh S.A.K. (2021). Evaluation of carcinogenic polyaromatic hydrocarbon levels in airborne particulates associated with long-term exposure throughout the COVID-19 pandemic in Makkah, Saudi Arabia. Int J Environ Res Publ Health.

[51] Moore P.S., Chang Y. (2010). Why do viruses cause cancer? Highlights of the first century of human tumour virology. Nat Rev Cancer.

[52] Zella D., Gallo R.C. (2021). Viruses and bacteria associated with cancer: an overview. Viruses.

[53] Lipsick J. (2021). A history of cancer research: tumor viruses. Cold Spring Harbor Perspect Biol.

[54] Heinsohn S., Szendroi M., Bielack S. (2009). Evaluation of SV40 in osteosarcoma and healthy population: a Hungarian-German study. Oncol Rep.

[55] Mendoza S.M., Konishi T., Miller C.W. (1998). Integration of SV40 in human osteosarcoma DNA. Oncogene.

[56] Peretti G., Torri G., Marinoni E.C. (1980). Experimental osteosarcoma in the mouse, induced by Moloney's murine sarcoma virus. Ital J Orthop Traumatol.

[57] Yamagata S., Yamagata T. (1984). FBJ virus-induced osteosarcoma contains type I, type I trimer, type III as well as type V collagens. J Biochem.

[58] Gimbel W., Schmidt J., Brack-Werner R. (1996). Molecular and pathogenic characterization of the RFB osteoma virus: lack of oncogene and induction of osteoma, osteopetrosis, and lymphoma. Virology.

[59] Acquaviva C., Bossis G., Ferrara P. (2002). Evasion from proteasomal degradation by mutated Fos proteins expressed from FBJ-MSV and FBR-MSV osteosarcomatogenic retroviruses. Biochem Pharmacol.

[60] Olson H.M., Capen C.C. (1977). Intratibial Moloney sarcoma virus-induced osteosarcoma in the rat: tumor incidence and pathologic evaluation. J Natl Cancer Inst.

[61] Finkel M.P., Biskis B.O., Jinkins P.B. (1966). Virus induction of osteosarcomas in mice. Science.

[62] Olson H.M., Capen C.C. (1977). Virus-induced animal model of osteosarcoma in the rat: morphologic and biochemical studies. Am J Pathol.

[63] Velupillai P., Sung C.K., Tian Y. (2010). Polyoma virus-induced osteosarcomas in inbred strains of mice: host determinants of metastasis. PLoS Pathog.

[64] Lee N.P., Chan C.M., Tung L.N. (2018). Tumor xenograft animal models for esophageal squamous cell carcinoma. J Biomed Sci.

[65] Shi J., Li Y., Jia R. (2020). The fidelity of cancer cells in PDX models: characteristics, mechanism and clinical significance. Int J Cancer.

[66] Blattmann C., Thiemann M., Stenzinger A. (2015). Establishment of a patient-derived orthotopic osteosarcoma mouse model. J Transl Med.

[67] Kohyama K., Sugiura H., Kozawa E. (2012). Antitumor activity of an interleukin-2 monoclonal antibody in a murine osteosarcoma transplantation model. Anticancer Res.

[68] Crnalic S., Häkansson I., Boquist L. (1997). A novel spontaneous metastasis model of human osteosarcoma developed using orthotopic transplantation of intact tumor tissue into tibia of nude mice. Clin Exp Metastasis.

[69] Owen L.N. (1969). Transplantation of canine osteosarcoma. Eur J Cancer.

[70] Goi K., Sugita K., Tezuka T. (2006). A successful case of allogeneic bone marrow transplantation for osteosarcoma with multiple metastases of lung and bone. Bone Marrow Transplant.

[71] Phulpin B., Marchettic N., Mansuyd L. (2012). Development of an osteosarcoma following dental extraction after allogeneic stem cell transplantation. Rev Laryngol Otol Rhinol.

[72] Pascoal S., Grissenberger S., Scheuringer E. (2021). Using zebrafish larvae as a xenotransplantation model to study Ewing sarcoma. Methods Mol Biol.

[73] Farese J.P., Fox L.E., Detrisac C.J. (2004). Effect of thalidomide on growth and metastasis of canine osteosarcoma cells after xenotransplantation in athymic mice. Am J Vet Res.

[74] Nilsson O.S., Lindholm T.S., Nilsonne U. (1982). Microdissection specimens of connective, chondrous, or bone tissue of human osteosarcomas and chondrosarcomas transplanted to athymic nude mice. Clin Orthop Relat Res.

[75] Zeng W.N., Yu Q.P., Wang D. (2021). Mitochondria-targeting graphene oxide nanocomposites for fluorescence imaging-guided synergistic phototherapy of drug-resistant osteosarcoma. J Nanobiotechnol.

[76] Chen F., Zhang Z., Shen R. (2022). Generation and characterization of patient-derived xenografts from patients with osteosarcoma. Tissue Cell.

[77] Schwartz A.L., Custis J.T., Harmon J.F. (2013). Orthotopic model of canine osteosarcoma in athymic rats for evaluation of stereotactic radiotherapy. Am J Vet Res.

[78] Siemionow M.Z., Kulahci Y., Bozkurt M. (2009). Composite tissue allotransplantation. Plast Reconstr Surg.

[79] Smith B.F., Migone F.K., Cox N.R. (2003). An *in utero* allotransplantation model of metastatic breast cancer in the cat. In Vivo.

[80] Okada S., Vaeteewoottacharn K., Kariya R. (2018). Establishment of a patient-derived tumor xenograft model and application for precision cancer medicine. *Chem Pharm Bull* (*Tokyo*).

[81] Guan G., Lu Y., Zhu X. (2015). CXCR4-targeted near-infrared imaging allows detection of orthotopic and metastatic human osteosarcoma in a mouse model. Sci Rep.

[82] Floersheim G.L., Bieri A., Chiodetti N. (1986). Xenografts in pharmacologically immunosuppressed mice as a model to test the chemotherapeutic sensitivity of human tumors. Int J Cancer.

[83] Guo Y., Rubin E.M., Xie J. (2008). Dominant negative LRP5 decreases tumorigenicity and metastasis of osteosarcoma in an animal model. Clin Orthop Relat Res.

[84] Wang Q. (2021). The cornerstone of translational research – selection of appropriate animal models. Biomater Transl.

[85] Ory B., Heymann M.F., Kamijo A. (2005). Zoledronic acid suppresses lung metastases and prolongs overall survival of osteosarcoma-bearing mice. Cancer.

[86] Goldstein S.D., Hayashi M., Albert C.M. (2015). An orthotopic xenograft model with survival hindlimb amputation allows investigation of the effect of tumor microenvironment on sarcoma metastasis. Clin Exp Metastasis.

[87] Robl B., Botter S.M., Pellegrini G. (2016). Evaluation of intraarterial and intravenous cisplatin chemotherapy in the treatment of metastatic osteosarcoma using an orthotopic xenograft mouse model. J Exp Clin Cancer Res.

[88] Igarashi K., Kawaguchi K., Kiyuna T. (2017). Effective metabolic targeting of human osteosarcoma cells *in vitro* and in orthotopic nude-mouse models with recombinant methioninase. Anticancer Res.

[89] Li Y., Liao Q., Li K. (2012). Knockdown of endothelin A receptor expression inhibits osteosarcoma pulmonary metastasis in an orthotopic xenograft mouse model. Mol Med Rep.

[90] Rainusso N., Man T.K., Lau C.C. (2011). Identification and gene expression profiling of tumor-initiating cells isolated from human osteosarcoma cell lines in an orthotopic mouse model. Cancer Biol Ther.

[91] Miretti S., Roato I., Taulli R. (2008). A mouse model of pulmonary metastasis from spontaneous osteosarcoma monitored *in vivo* by luciferase imaging. PLoS One.

[92] Wang R., Liu X. (2020). Epigenetic regulation of prostate cancer. Genes Dis.

[93] Fardi M., Solali S., Farshdousti Hagh M. (2018). Epigenetic mechanisms as a new approach in cancer treatment: an updated review. Genes Dis.

[94] Wang Z.Q., Liang J., Schellander K. (1995). *c-fos*-induced osteosarcoma formation in transgenic mice: cooperativity with *c-jun* and the role of endogenous *c-fos*. Cancer Res.

[95] Huang P., McKee T.D., Jain R.K. (2005). Green fluorescent protein (GFP)-expressing tumor model derived from a spontaneous osteosarcoma in a vascular endothelial growth factor (VEGF)-GFP transgenic mouse. Comp Med.

[96] Lu Y., Gitelis S., Lei G. (2014). Research findings working with the p53 and Rb1 targeted osteosarcoma mouse model. Am J Cancer Res.

[97] Entz-Werlé N., Choquet P., Neuville A. (2010). Targeted *Apc;Twist* double-mutant mice: a new model of spontaneous osteosarcoma that mimics the human disease. Transl Oncol.

[98] Ren S.S., Yuan F., Liu Y.H. (2015). Effect of p27 gene combined with Pientzehuang on tumor growth in osteosarcoma-bearing nude mice. Chin J Integr Med.

[99] Molyneux S.D., Di Grappa M.A., Beristain A.G. (2010). Prkar1a is an osteosarcoma tumor suppressor that defines a molecular subclass in mice. J Clin Invest.

[100] Obana K., Yang H.W., Piao H.Y. (2003). Aberrations of p16INK4A, p14ARF and p15INK4B genes in pediatric solid tumors. Int J Oncol.

[101] Lv S.Y., Cui B., Yang Y. (2019). Spexin/NPQ induces FBJ osteosarcoma oncogene (Fos) and produces antinociceptive effect against inflammatory pain in the mouse model. Am J Pathol.

[102] Walia M.K., Castillo-Tandazo W., Mutsaers A.J. (2018). Murine models of osteosarcoma: a piece of the translational puzzle. J Cell Biochem.

[103] Arlt M.J.E., Banke I.J., Walters D.K. (2011). LacZ transgene expression in the subcutaneous Dunn/LM8 osteosarcoma mouse model allows for the identification of micrometastasis. J Orthop Res.

[104] Scharf V.F., Farese J.P., Siemann D.W. (2014). Effects of aurothiomalate treatment on canine osteosarcoma in a murine xenograft model. Anti Cancer Drugs.

[105] Yu Z., Sun H., Fan Q. (2009). Establishment of reproducible osteosarcoma rat model using orthotopic implantation technique. Oncol Rep.

[106] Bertin H., Guilho R., Brion R. (2019). Jaw osteosarcoma models in mice: first description. J Transl Med.

[107] Withrow S.J., Powers B.E., Straw R.C. (1991). Comparative aspects of osteosarcoma. Dog versus man. Clin Orthop Relat Res.

[108] Giner F., López-Guerrero J.A., Machado I. (2015). The early stages of tumor angiogenesis in human osteosarcoma: a nude mice xenotransplant model. Virchows Arch.

[109] Marques da Costa M.E., Daudigeos-Dubus E., Gomez-Brouchet A. (2018). Establishment and characterization of *in vivo* orthotopic bioluminescent xenograft models from human osteosarcoma cell lines in Swiss nude and NSG mice. Cancer Med.

[110] Miao M., Masengere H., Yu G. (2021). Reevaluation of NOD/SCID mice as NK cell-deficient models. Biomed Res Int.

[111] Oldham R.J., Mockridge C.I., James S. (2020). FcγRII (CD32) modulates antibody clearance in NOD SCID mice leading to impaired antibody-mediated tumor cell deletion. J Immunother Cancer.

[112] Joseph S., Singal D.P., Ludwin D. (1989). Comparison of immune responses in mice after transfusions from single or multiple H-2 donors. Transplant Proc.

[113] Mohseny A.B., Hogendoorn P.C.W., Cleton-Jansen A.M. (2012). Osteosarcoma models: from cell lines to zebrafish. Sarcoma.

[114] Mohseny A.B., Xiao W., Carvalho R. (2012). An osteosarcoma zebrafish model implicates Mmp-19 and Ets-1 as well as reduced host immune response in angiogenesis and migration. J Pathol.

[115] Allen T.A., Cullen M.M., Hawkey N. (2021). A zebrafish model of metastatic colonization pinpoints cellular mechanisms of circulating tumor cell extravasation. Front Oncol.

[116] Vimalraj S., Subramanian R., Saravanan S. (2021). MicroRNA-432-5p regulates sprouting and intussusceptive angiogenesis in osteosarcoma microenvironment by targeting PDGFB. Lab Invest.

[117] Pautke C., Schieker M., Tischer T. (2004). Characterization of osteosarcoma cell lines MG-63, Saos-2 and U-2 OS in comparison to human osteoblasts. Anticancer Res.

[118] Mu X., Isaac C., Greco N. (2013). Notch signaling is associated with ALDH activity and an aggressive metastatic phenotype in murine osteosarcoma cells. Front Oncol.

[119] Yuan J., Ossendorf C., Szatkowski J.P. (2009). Osteoblastic and osteolytic human osteosarcomas can be studied with a new xenograft mouse model producing spontaneous metastases. Cancer Invest.

[120] McCauley H.A., Guasch G. (2013). Serial orthotopic transplantation of epithelial tumors in single-cell suspension. Methods Mol Biol.

[121] Lipsitt A., Hensch N.R., Moreno-Campos R. (2021). Zebrafish tumor graft transplantation to grow tumors *in vivo* that engraft poorly as single cell suspensions. Zebrafish.

[122] Wu H., He Z., Li X. (2021). Efficient and consistent orthotopic osteosarcoma model by cell sheet transplantation in the nude mice for drug testing. Front Bioeng Biotechnol.

[123] Akimoto J., Takagi S., Nakayama M. (2016). Transplantation of cancerous cell sheets effectively generates tumour-bearing model mice. J Tissue Eng Regen Med.

[124] Handal J.A., Schulz J.F., Florez G.B. (2013). Creation of rabbit bone and soft tissue tumor using cultured VX2 cells. J Surg Res.

[125] Hildreth B.E., Palmer C., Allen M.J. (2020). Modeling primary bone tumors and bone metastasis with solid tumor graft implantation into bone. J Vis Exp.

[126] Varshney J., Scott M.C., Largaespada D.A. (2016). Understanding the osteosarcoma pathobiology: a comparative oncology approach. Vet Sci.

[127] Saalfrank A., Janssen K.P., Ravon M. (2016). A porcine model of osteosarcoma. Oncogenesis.

[128] Simpson S., Dunning M.D., de Brot S. (2017). Comparative review of human and canine osteosarcoma: morphology, epidemiology, prognosis, treatment and genetics. Acta Vet Scand.

[129] van Leeuwen I.S., Cornelisse C.J., Misdorp W. (1997). P53 gene mutations in osteosarcomas in the dog. Cancer Lett.

[130] Johnson A.S., Couto C.G., Weghorst C.M. (1998). Mutation of the p53 tumor suppressor gene in spontaneously occurring osteosarcomas of the dog. Carcinogenesis.

[131] Meazza C., Scanagatta P. (2016). Metastatic osteosarcoma: a challenging multidisciplinary treatment. Expert Rev Anticancer Ther.

[132] Du L., Xu W.T., Fan Q.M. (2012). Tumorigenesis and spontaneous metastasis by luciferase-labeled human xenograft osteosarcoma cells in nude mice. Chin Med J.

[133] Rousseau J., Escriou V., Perrot P. (2010). Advantages of bioluminescence imaging to follow siRNA or chemotherapeutic treatments in osteosarcoma preclinical models. Cancer Gene Ther.

[134] Ruiz M.C., Resasco A., Di Virgilio A.L. (2019). *In vitro* and *in vivo* anticancer effects of two quinoline-platinum(II) complexes on human osteosarcoma models. Cancer Chemother Pharmacol.

[135] Guder W.K., Hartmann W., Trautmann M. (2020). Analysis of drug sensitivity of human high-grade osteosarcoma in a chick chorioallantoic membrane (CAM) model: a proof of principle study. BMC Res Notes.

[136] Rao D.D., Jay C., Wang Z. (2016). Preclinical justification of pbi-shRNA EWS/FLI1 lipoplex (LPX) treatment for Ewing's sarcoma. Mol Ther.

